# Objective Assessment of Physiologic Alterations Associated With Hemodynamically Significant Patent Ductus Arteriosus in Extremely Premature Neonates

**DOI:** 10.3389/fped.2021.648584

**Published:** 2021-02-25

**Authors:** Aparna Patra, Pratibha S. Thakkar, Majd Makhoul, Henrietta S. Bada

**Affiliations:** ^1^Division of Neonatology, Department of Pediatrics, Kentucky Children's Hospital, University of Kentucky, Lexington, KY, United States; ^2^Division of Pediatric Cardiology, Department of Pediatrics, Kentucky Children's Hospital, University of Kentucky, Lexington, KY, United States

**Keywords:** hsPDA, preterm, physiologic, echocardiogram, near-infrared spectroscopy, electrical cardiometry

## Abstract

Delay in closure of ductus arteriosus in postnatal life may lead to serious consequences and complications in an extremely premature neonate secondary to hemodynamic alterations in regional blood flow pattern in various organs. Despite the widespread recognition amongst neonatologists to identify a hemodynamically significant patent ductus arteriosus (hsPDA) early in the postnatal course, there is lack of consensus in its definition and thus the threshold to initiate treatment. Echocardiographic assessment of PDA shunt size and volume combined with neonatologists' impression of clinical significance is most frequently used to determine the need for treatment of PDA. Common clinical signs of hsPDA utilized as surrogate for decreased tissue perfusion may lag behind early echocardiographic signs. Although echocardiogram allows direct assessment of PDA shunt and hemodynamic alterations in the heart, it is limited by dependence on pediatric cardiologist availability, interobserver variation and isolated time point assessment. Electrical cardiometry (EC) is a non-invasive continuous real time measurement of cardiac output by applying changes in thoracic electrical impedance. EC has been validated in preterm newborns by concomitant transthoracic echocardiogram assessments and may be beneficial in studying changes in cardiac output in premature newborns with hsPDA. Alterations in perfusion index derived from continuous pulse oximetry monitoring has been used to study changes in cardiac performance and tissue perfusion in infants with PDA. Near infrared spectroscopy (NIRS) has been used to objectively and continuously assess variations in renal, mesenteric, and cerebral oxygen saturation and thus perfusion changes due to diastolic vascular steal from hsPDA in preterm neonates. Doppler ultrasound studies measuring resistive indices in cerebral circulation indicate disturbance in cerebral perfusion secondary to ductal steal. With recent trends of change in practice toward less intervention in care of preterm newborn, treatment strategy needs to be targeted for select preterm population most vulnerable to adverse hemodynamic effects of PDA. Integration of these novel ways of hemodynamic and tissue perfusion assessment in routine clinical care may help mitigate the challenges in defining and targeting treatment of hsPDA thereby improving outcomes in extremely premature neonates.

## Introduction

Ductus arteriosus is an important and necessary structure for the fetus. It allows communication between the pulmonary artery and descending aorta during fetal life facilitating majority of right ventricular output to bypass the pulmonary vascular bed and enter the descending aorta supporting systemic oxygenation. Delay in its postnatal closure as commonly encountered in extremely premature infants born <28 weeks gestation (1) can result in failure of circulatory adaptation leading to hemodynamic alterations in regional blood flow pattern in multiple organs and serious complications in both early and late postnatal course. A recent retrospective study examining the incidence of patent ductus arteriosus (PDA) in extremely premature babies found highest rate of 93% in infants born at 23–24 weeks, 64% in 25–26 weeks, and 21% in 27–28 weeks of gestation (2).

The clinical consequences depend on the degree of left-to-right shunting through the PDA and ductal steal. The increase in pulmonary blood flow in the setting of prematurity can lead to pulmonary edema, pulmonary hemorrhage, respiratory deterioration, pulmonary hypertension, left atrial and ventricular overload and dilation followed by left ventricular dysfunction (3, 4). Diminished gastrointestinal, renal, and cerebral blood flow secondary to PDA has been historically linked to increased risk of necrotizing enterocolitis, isolated intestinal perforation; acute kidney injury and intraventricular hemorrhage, respectively (5). The above adverse hemodynamic effects have led to the widespread recognition of identifying a hemodynamically significant PDA (hsPDA), however there is lack of consensus in its definition. Previously published trials used variable definitions or lacked specific treatment criteria or included more mature gestational ages and hence the optimal threshold to identify the infants at the highest risk of the above adverse sequelae is not well-delineated (5–7).

Persistent patency of hsPDA is indisputably associated with increased morbidity and mortality in premature neonates. However, there is debate whether early medical or surgical closure of PDA improves outcomes. Hence, in the last decade, approach to PDA has shifted from early treatment to watchful waiting for spontaneous closure (8). A recent pilot exploratory trial showed similar ductal persistence rates at discharge when early PDA intervention strategy was compared to targeted therapy in presence of persistent hsPDA at ≥7days of life. Extremely premature neonates of gestational age ≥26 weeks managed with routine early treatment approach in the first week of life had higher incidence of late-onset sepsis and longer duration to achieve full enteral feeds with no advantage of reducing morbidities or ductal ligation incidence at discharge. Selective waiting and targeted approach to PDA management although reasonable in some extremely premature babies, may lead to prolonged exposure to ductal shunt and its associated detrimental effects particularly in gestational age <26 weeks (9). Data from Pediatric Hospital Information System demonstrated a temporal association between declining pharmacotherapy for PDA and unadjusted increases in long term pulmonary and neurological impairment among extremely low birth weight neonates (10). Therefore, in the current era when neonates born as early as 22 weeks are surviving, neonatologists find themselves vacillating and wavering in their decision of management of PDA.

Currently echocardiographic measurements of a PDA are considered as gold standard for assessing the magnitude and relevance of left to right shunting in premature newborns (11). Many authors have established echocardiographic criteria for hemodynamic significance of a PDA (11–13). Echocardiography gives information of a single time point and is limited by dependence on availability of a pediatric cardiologist and interobserver variation. Also, the vulnerability of the patient to the PDA is incompletely assessed by an echocardiogram with regards to disturbances in organ blood flow and adverse hemodynamic effect in other organs which is critical in assessing need for treatment. The clinical signs of alteration in organ perfusion such as widening pulse pressure, decreased mean blood pressure, fluctuations in heart rate, hypoxemia, oliguria, worsening metabolic acidosis and increasing respiratory support lack clinical sensitivity (14) and may lag behind the early hemodynamic effects noted on echocardiography (15, 16). Continuous assessment and accurate interpretation of the variations and fluctuations in physiological parameters associated with hsPDA can aid in timely diagnosis and management. Current biomedical sophistication and advancement allows processing of physiologic signals in preterm neonate caused by failed circulatory adaptation and hemodynamic effects of PDA. In this review, we discuss the various novel techniques that may complement or improve the assessment of hemodynamic alterations in PDA; these methods have been examined and validated in clinical studies which can further our knowledge about the hemodynamic vulnerability of a neonate with hsPDA.

### Arterial Blood Pressure and the Dilemma of Systemic Hypotension in hsPDA

Arterial blood pressure (BP) is one of the commonly used physiologic signals for evaluating hemodynamic status associated with any clinical condition. Invasive arterial catheter is the gold standard for measuring arterial BP in the initial course of premature newborn followed by oscillometric monitoring. There are technical concerns with inaccurate and under or over estimation of BP with either methods in this population (17, 18) however its low cost, uncomplicated technique and feasibility in small sized premature newborns makes it widely used in the neonatal intensive care unit. Normogram for mean invasive BP based on gestational and postnatal age has been established in neonates (19) and most commonly practiced hypotension criteria is mean arterial pressure less than infant's postmenstrual age (20). Extremely premature infants are predisposed to low mean blood pressure in immediate postnatal period due to myocardial and autonomic immaturity which may be further exacerbated by foramen ovale or ductal shunting. Blood pressure changes secondary to hsPDA may initially manifest as decrease in diastolic BP relative to systolic BP and eventually followed by reduction in both systolic and diastolic BP (21, 22). However, BP in sick ventilated preterm newborns does not always correlate with central blood flow, organ perfusion and oxygen delivery and may be associated with normal or high cardiac output (23–25) making sole reliance on BP for assessment of hemodynamic magnitude of PDA not useful. Also threshold for intervention for hypotension in preterm population varies widely amongst neonatologists (26) due to variable evidence about long term neurodevelopmental effects with hypotension tolerance vs. treatment (27–29). Further, as mentioned above, changes in BP, clinical signs of hypotension and compromised systemic perfusion may have a later onset compared to early signs of ductal steal on echocardiogram. Thus, a more precise and individualized approach for early determination of hemodynamic magnitude of PDA is required in the truly vulnerable micro preemie.

### Neonatologist Performed Targeted Echocardiography

Echocardiography is the gold standard of predicting and diagnosing hemodynamic significance of PDA. hsPDA may go clinically unnoticed in initial postnatal course of a preterm neonate as clinicals signs of hsPDA lag behind echocardiographic measurements by mean of 2 days (16). Performance of bedside-targeted echocardiography by neonatologists (also known as functional echocardiography fECHO) is gaining popularity in contemporary neonatal clinical practice to assist in early diagnosis and enhance medical decision making although its use continues to be limited in the United States (30).

The European special interest group for Neonatologist Performed Echocardiography (31) describes specific parameters to objectively quantify the hemodynamic magnitude and relevance of PDA. Transductal dimension (measured at the narrowest site on the pulmonary end), shunt direction, ratio of systolic to diastolic shunt flow velocity and transductal pressure gradient are important determinants of shunt volume. Higher transductal shunt volumes are associated with increased pulmonary and decreased systemic perfusion. Further comprehensive objective measurements of pulmonary over circulation can be estimated from left ventricular output and parameters of left sided volume (left atrium/aortic root ratio, left pulmonary artery diastolic flow velocity) and pressure load that possibly explains associated morbidities such as pulmonary hemorrhage, high need for respiratory support, and later risk of bronchopulmonary dysplasia. Large volume of interatrial shunts may artificially lower left atrium/aortic root ratio or left ventricular end diastolic volume and should be carefully evaluated (32). The impact of systemic hypoperfusion secondary to ductal steal can be assessed by the flow direction of forward, absent or reverse in descending aorta, celiac trunk and middle cerebral artery during diastole. Transductal diameter ≥1.5 mm in initial 31 h of life, first appearance of doppler pulsatile ductal flow pattern, and ratio of systolic to diastolic ductal flow velocity >1.9 in the first 48 h are the parameters with high sensitivity and specificity for predicting patency of ductus arteriosus (32–37). Ductal diameter and left ventricular output are frequently used to determine need of therapeutic intervention for hsPDA (34, 38). Repeated assessment of above parameters on fECHO and monitoring trends can help evaluate and establish diagnosis of hsPDA.

Various scoring systems, incorporating measurements from fECHO have been published to standardize the assessment of hemodynamic severity of the PDA (12, 38). These scoring systems utilizing fECHO parameters have been seen to decrease risk of in hospital mortality (39) and or pulmonary hemorrhage (39, 40) or chronic lung disease (38) in preterm infants treated for hsPDA. Additionally, use of fECHO during treatment may help in monitoring response to medication minimizing drug doses and adverse effects from pharmacotherapy (41, 42). Many of these fECHO measurements are subjective to interobserver variations (43) and the need for additional prolonged training (44) of neonatologists have limited its use in clinical settings despite high value in clinical decision making. Repeated assessment of discussed parameters on fECHO and monitoring trends rather than following absolute cut off values may be beneficial in evaluation of hsPDA.

### Electrical Cardiometry—An Adjunct to Echocardiogram With Continuous Data

Assessment of the hemodynamic status particularly cardiac function is crucial for understanding the hemodynamic impact of hsPDA in preterm neonates. In neonatal practice, invasive blood pressure is routinely and continuously monitored in initial days of life for premature infants followed by intermittent non-invasive oscillometric blood pressure monitoring. Assessment of cardiac output (CO) by interpretation of indirect variables like blood pressure can be unreliable in premature and sick neonates (45). Other reliable invasive method of measuring cardiac output utilizing thermodilution catheter is not clinically feasible in extremely premature neonates and the limitations of sporadic information from technically demanding and operator dependent echocardiogram has been previously discussed.

Electrical Cardiometry (EC, also known as electrical velocimetry) has been proposed as a safe, well-tolerated, practical, non-invasive, continuous method of measurement of cardiac output that is reproducible in neonates of various gestational maturity, weight and body surface area (46, 47). EC works on the principle of electrical bioimpedance which is calculated based on the variation in aortic blood flow using surface adhesive electrodes placed on the neck or scapular area and thorax (48). Forward flow during systole causes unidirectional configuration of erythrocytes parallel to blood flow which results in better conductivity. During diastolic filling stage, there is abrupt cessation of this blood flow leading to haphazard arrangement of erythrocytes which increases the impedance and dampens the conductivity. This difference in impedance creates a waveform which is utilized to measure blood velocity, stroke volume and cardiac output (49, 50). Clinical feasibility and application of EC has been studied in animal models (51), adult humans (52), pediatric congenital heart disease patients (53), and in more recent years in term and preterm neonates (47, 48, 54–56).

Authors have established the validity of EC by demonstrating that stroke volume and cardiac output measured by EC are comparable with echocardiogram with estimated similar bias, precision and acceptable percentage error in neonates (47, 54, 57). These studies investigated preterm infants with hsPDA (variable definition based on ductal size and left atrium aortic root ratio) and found that EC cardiac output measurements were clinically interchangeable with echocardiogram findings. Another recent study utilized EC as one of the parameters for early prediction of hsPDA in a cohort of neonates <32 weeks gestation. The authors found that a trend of increased left ventricular output on EC at 6 h of life correlated with hsPDA requiring pharmacotherapy (7).

Despite the accumulating evidence of clinically advantageous and acceptable values between EC and echocardiogram in hemodynamic assessment of hsPDA, it is important to note that reference values of premature neonate cardiac function on EC is yet under research and not clinically established (56). Also larger percentage of error in EC measurements is a concern in babies on invasive mechanical ventilation particularly on high frequency ventilation which is commonly used in clinical practice for critically sick extremely premature neonates (54, 56). Another limitation of EC in hemodynamic assessment of hsPDA is it fails to measure preload or intravascular volume (56) which is important to know to prevent injudicious volume expansion in these patients who are at high risk of intracranial hemorrhage. The integration of EC in clinical neonatology hence is currently limited to trending cardiac function and the hemodynamic impact rather than making clinical decision on absolute values.

### Near-Infrared Spectroscopy and Regional Oxygenation

One of the important aspects of hemodynamic disturbance in hsPDA is the “diastolic run-off” that leads to attenuated blood flow to various peripheral organs (58). Oxygen delivery to tissues depends on blood flow and oxygen content which is reduced in these circumstances resulting in decreased regional oxygen saturation (RSO_2_) in multiple organs particularly cerebral (cRSO_2_), renal (rRSO_2_), and splanchnic beds (sRSO_2_) necessitating physiological increase in fractional tissue oxygen extraction (FTOE) to maintain tissue oxygen requirement in premature neonates. Use of NIRS has progressed from being a research tool to clinical bedside device for non-invasive, continuous assessment of this changing oxygen dynamics in presence of hsPDA (59–61) as well as other clinical applications in neonatology.

NIRS technology uses light from infrared spectrum with wavelength between 700 and 1,000 nm. As light passes through the tissue, it is partly absorbed, reflected and scattered. Probes comprising of transmitter and receiver optodes applied on skin, detects the reflected portion of light which varies depending on the absorptive capacity of the molecules present. NIRS measures oxygen saturation by assessing the relative amount of oxyhemoglobin and deoxyhemoglobin content in the tissue (62). Due to deeper penetrance of infrared light in the tissue, signal for NIRS is derived from all vascular beds with major contribution from venous compartment (75%) followed by arterial and capillary (20 and 5% (63) which makes NIRS measurements specific and reflective of regional tissue oxygen balance by calculating oxygen delivery and consumption (62). Unlike pulse oximetry that calculates arterial oxygen saturation, NIRS provides information on alteration in tissue oxygen consumption which may be an early marker of perfusion injury (62, 64) making it complementary to pulse oximetry.

Specialized flexible mini-sensors as well as smaller sized adult sensors are available for use in neonates (65) that can be applied on the skin surface on forehead, flank region and periumbilical region to assess cRSO_2_, rRSO_2_, and sRSO_2_, respectively (66). Reference values of various sensors used in neonates have been published (67). Various authors have published reference ranges for cRSO_2_ starting from birth through initial weeks of life and advancing postnatal age for both term (68–70) and preterm infants (71, 72). Gestational age stratified normative values for cRSO_2_ in preterm neonates <32 weeks have been validated for use in first 72 h of life (65).

NIRS monitoring can aid in early recognition of hsPDA by identifying the magnitude of compromise in renal, cerebral and mesenteric circulation. Studies have reported decreased rRSO_2_ level of 61 ± 3% is associated with hsPDA with further decreased level of <43% predicting the need for PDA closure (73, 74). Positive correlation was noted between ductal size and persistently low cRSO_2_ levels in infants with ductal patency irrespective of its hemodynamic relevance (75). Prolonged exposure to lower cerebral oxygenation as seen in infants with persistent patency of ductus requiring surgical closure is associated with impaired brain growth and poor neurodevelopmental outcomes (76, 77).

Additional utility of NIRS may be in monitoring of tissue perfusion during and following pharmacotherapy or surgical intervention for hsPDA. Studies have shown prompt improvement in cerebral and mesenteric perfusion after pharmacological and surgical closure of PDA (78, 79). Brief improvements in end-organ perfusion may be followed by reduction in regional blood flow in post ductal ligation patients owing to decreased left ventricular cardiac output. Continuous and concurrent monitoring of regional oxygenation in brain and kidneys perioperatively may enable clinicians to diagnose post ligation cardiac syndrome before the onset of clinical symptoms related to decreased left ventricular output (80).

### Perfusion Index and Alteration in Peripheral Perfusion

There is growing controversial evidence in the neonatal literature that arterial blood pressure may not be a reliable indicator for estimation of peripheral perfusion in a premature neonate (81, 82). Early identification of compromised peripheral perfusion in a premature neonate with PDA is important for clinical decision making about its hemodynamic impact. Goal of minimizing handling in such patient cohort makes serial echocardiogram difficult to perform.

Peripheral pulse-oximetry is a routinely and readily applied monitoring device for premature neonates which does not require any special preparation or handling. Pulse oximeters generate a photoplethysmography waveform at infrared and red wavelengths. Perfusion index (PI) is a non-invasive method of objectively measuring peripheral perfusion from the plethysmography signal generated by pulse oximeters and represents the ratio of light absorbed by pulsatile elements such as arteries and non-pulsatile elements such as blood in venous or capillary bed, bone, connective tissue etc. (83, 84). PI has been shown to be an objective indicator of hemodynamic alterations and correlate with peripheral perfusion, cardiac output, and stroke volume (84, 85).

Studies have examined PI values in healthy and sick term neonates (86) as well as preterm infants (87) with an aim to derive normative values. Authors have also demonstrated PI values in first week of life in babies with ductal dependent circulation (88). Difference in PI measurements between right upper extremity (pre ductal) and either of lower extremities (post ductal) may be indicative of poor post ductal perfusion and thus suggestive of presence of hsPDA (89). Changes in PI can also be affected by changes in skin temperature at monitoring site (84).

Decrease in superior vena cava (SVC) flow may be a novel marker of compromised systemic circulation in neonates with hsPDA (90). PI has been shown to correlate with SVC flow in premature neonates and has been associated with volume responsiveness in sick neonates (91, 92). Even though increased pulsatile component from bounding pulses in hsPDA can falsely increase PI more than true perfusion, authors found the correlation of PI with SVC flow remains valid discerning the hemodynamic impact of the PDA in a cohort of premature newborns born <32 weeks of gestation (91).

Other authors reported that the PI of the lower extremities (post-ductal) is decreased compared to the right arm (pre-ductal) in preterm infants with hsPDA which measured by delta PI of a certain cut off value strongly correlated with the echocardiographic diagnosis of hsPDA (93). In another study that reviewed the relation of PI and PDA, authors noted that delta PI, delta PI variability, and mean pre-ductal PI measured 4 h prior to echocardiography reliably detected PDA in preterm infants (94). PI is a simple, continuous dynamic measure of peripheral perfusion which may be a useful adjunct in helping identify preterm babies vulnerable to circulatory adversities of a hsPDA.

### Other Biomarkers for Early Detection

The review of physiologic alterations secondary to hsPDA would be incomplete without discussion of certain biomarkers that have been studied and incorporated in clinical practice for assessment of hemodynamic significance of PDA. Cardiac peptides such as B-type (Brain) natriuretic peptide (BNP) and N-terminal pro-BNP(NT-proBNP) have been studied extensively for early detection as well as monitoring therapy of hsPDA. NT-pro BNP is a marker of heart failure that increases due to ventricular volume and pressure overload in hsPDA. Normative values for this peptide in plasma in first week of life has been studied at various gestational ages, with lower normal values noted in term infants compared to preterms (95). Various authors have examined the association of plasma BNP and NT-proBNP levels with echocardiographic parameters of hemodynamic significance (96, 97). Serum cut off values have been investigated for early detection of hemodynamic significance by day of life 3 (98), predicting probability of spontaneous ductal closure (99) and developing a scoring system for PDA and associated outcomes (100). Further non-invasive avenues have been explored recently with study of urinary NT-proBNP to predict ductal diameter (101) and non-response to pharmacotherapy (102, 103). Patients with medically treated hsPDA can have persistently elevated urinary NT-proBNP levels and NT-proBNP/creatinine ratio on day of life 14 indicative of cardiac strain secondary to residual volume overload from ductal shunt beyond its pharmacological closure (104).

Alterations of organ perfusion associated with hsPDA can lead to production of ischemia modified albumin, serum levels of which were found to be elevated in cohort of preterm neonates with hsPDA (105). Relationship of end tidal carbon monoxide measured non-invasively in exhaled gas and hsPDA has been studied in preterm infants as carbon monoxide is a known regulator of muscle relaxation and may be implicated in the physiologic closure of ductus arteriosus. Higher end tidal carbon monoxide levels early after birth was associated with hsPDA secondary to relaxant effect of carbon monoxide on ductal muscular tone (106).

Elevated nucleated red blood cell (NRBC) count at birth is related to chronic uterine hypoxia which may predispose to PDA. A recent prospective study of preterm infants, showed differences in absolute NRBC count with ductal size and hemodynamic significance of PDA as measured by echocardiogram. The authors proposed cut off absolute NRBC levels to predict hsPDA (107).

There is ambiguous data on association of thrombocytopenia with hsPDA and its influence on response to treatment. Presence of low platelet count and high platelet distribution width in preterm neonates is shown to be associated with hsPDA (108). There is report of neonates with platelet dysfunction (as measured by platelet function analyzer-100) to have prolonged ductal patency as detected on echocardiography in first 72–96 h of life (109).

## Conclusion

**Figure 1:**
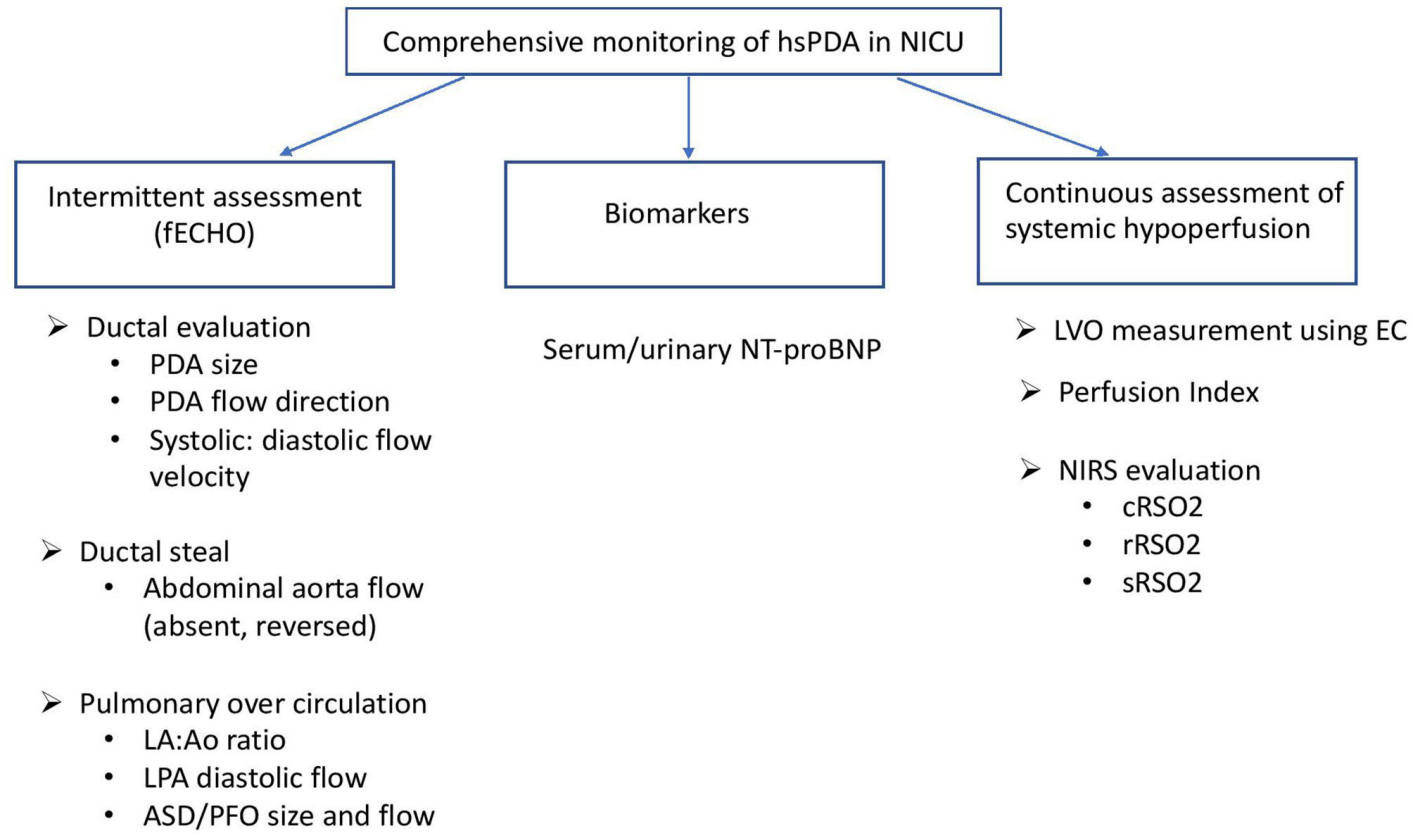
Monitoring of hsPDA in the NICU. fECHO, functional echocardiogram; LA, left atrium; Ao, aortic root; LPA, left pulmonary artery; ASD, atrial septal defect; PFO, patent foramen ovale; LVO, left ventricular output; EC, electrical cardiometry; NIRS, near infrared spectroscopy; cRSO_2_, cerebral regional oxygen saturation; rRSO_2_, renal regional oxygen saturation; sRSO_2_, splanchnic regional oxygen saturation.

The definition of hsPDA and the population it applies to, continues to develop and unfold with accumulation of further evidence of novel ways of assessment and outcomes. This review discusses the role of various continuous modes of tapping and analyzing physiologic variables resulting from a hsPDA beyond the cardiologist's echocardiogram as summarized in Figure 1. Such continuous monitoring allows better and earlier understanding of the magnitude of ductal shunting by assessing the vulnerability of organs at risk of over-circulation or hypoperfusion. There is emerging evidence that routine and widespread treatment for PDA in preterm infants is not necessarily associated with improved outcomes (110). However, there is a cohort of extremely premature infants of the most immature gestation with need for precise and deliberate approach for early identification of severe hemodynamic disturbance associated with hsPDA in the first week of life. Further research for risk stratification to identify this select cohort of neonates is needed. Advantages and limitations of various modalities for ductal hemodynamic monitoring has been summarized in Table 1. The continuous complementary data for monitoring of this hemodynamic disturbance is encouraging, however prior to integration in clinical care, the question whether assessment of these parameters and resulting interventions lead to enhancement in short- or long-term outcomes will need to be answered. Also, with most of these parameters lacking established reference values in preterm neonates, currently they are useful at the bedside primarily for trending of physiologic alterations specific to a patient rather than indicating treatment threshold.

**Table 1:** Advantages and limitations of methods for bedside hemodynamic monitoring of PDA in Neonatal ICU.

**Monitoring modality**	**Advantages**	**Disadvantages**
Arterial blood pressure	• Widely used • Low cost • Accurate blood pressure measurement	• Invasive • Does not measure organ perfusion/oxygen delivery • Detects late sign of hsPDA
Targeted echocardiography	• Early assessment of PDA hemodynamics • Established reference values available • Independent of cardiologist's availability	• Interobserver variation • Need for extensive training for neonatologists
Electrical cardiometry	• Non-invasive • Continuous monitoring of cardiac function • Precise values comparable to echocardiography • Assess early signs of hsPDA (change in LVCO)	• Evolving reference values in preterm • Discrepancy in neonates on invasive high frequency ventilation
NIRS	• Continuous monitoring • Non invasive • Assessment of peripheral organ perfusion • Multiple site assessment • Trend monitoring	• Evolving site specificreference values in preterm • Lack of absolute threshold values
Perfusion index	• Widely available • Continuous monitoring • Non-invasive • Correlates with change in organ blood flow • Trend monitoring	• Lack of absolute threshold values
Biomarkers NT-proBNP nucleated RBC count platelet count	• Widely available • Correlation between serum and urinary NT-proBNP	• Abnormal values are not specific to PDA

*hsPDA, hemodynamically significant patent ductus arteriosus; LVCO, left ventricular cardiac output; NT-proBNP, N-terminal B-type natriuretic peptide; NIRS, near infrared spectroscopy*.

## Author Contributions

AP conceived the idea for the article, reviewed the current literature, and wrote the manuscript. PT reviewed the literature and assisted in writing various sections of the manuscript. MM and HB critically reviewed the manuscript. All authors reviewed the final manuscript.

## Conflict of Interest

The authors declare that the research was conducted in the absence of any commercial or financial relationships that could be construed as a potential conflict of interest.
